# Sustainable bio-based solid phase extraction adsorbent for the determination of various types of organic compounds

**DOI:** 10.55730/1300-0527.3637

**Published:** 2023-11-27

**Authors:** Mustafa Zafer ÖZEL, James H. CLARK, Avtar S. MATHARU

**Affiliations:** 1 Department of Clinical, Pharmaceutical and Biological Sciences, School of Life and Medical Sciences, University of Hertfordshire, College Lane, Hatfield, UK; 2 Department of Chemistry, Green Chemistry Centre of Excellence, University of York, York, UK

**Keywords:** Adsorbent, Starbon A800, solid phase extraction, gas chromatography, UV-visible spectroscopy

## Abstract

A sustainable, bio-based, mesoporous material, Starbon A800, was explored for use as an adsorbent in solid phase extraction (SPE). A solution containing seven nitrosamines was first used as a standard to optimise conditions for extraction efficiency with Starbon A800. After optimising conditions, 25 compounds of varying polarity (terpenes, phenolics, pesticides, PAHs, amines, and nitrosamines) were extracted with SPE using either Starbon® A800, C18 or Porous Graphitic Carbon (PGC) as the adsorbent, for comparison purposes. At the same time, 3 different elution solvents (heptane, dichloromethane, and ethanol) were used for each type of adsorbent. Hansen solubility parameters can be used to choose an appropriate elution solvent for the selected SPE adsorbent. The best average SPE recoveries found for the 25 various compounds were 83%, 79%, and 65% using Starbon A800, PGC, and C18 adsorbents respectively and these had dichloromethane as the elution solvent. The identification and quantification of components was carried out using UV-visible spectroscopy, two-dimensional gas chromatography (GCxGC) with time of flight/mass spectrometry (TOF/MS) or a nitrogen chemiluminescence detector (NCD). The optimized method was successfully applied to extract volatile organic compounds from red wine and tap water using Starbon A800. Starbon A800 was shown to be a promising, low-cost, green, scalable, alternative adsorbent for the extraction of various types of organic compounds of a wide range of polarities using SPE.

## 1. Introduction

Complex mixtures of differing structures and polarity are difficult to separate and identify. Sample preparation is crucial in environmental, biomedical, food, and pharmaceutical analysis and often involves tedious and time-consuming procedures. Sample preparation typically takes 80% of the total analysis time [1]. The classical separation methods are distillation, extraction, or fractionation techniques.

Solid Phase Extraction (SPE) was introduced in the early 1970s and avoids or minimizes the disadvantages of liquid-liquid extraction (LLE) [2]. The main objectives of SPE are the removal of interfering compounds, pre-concentration and/or fractionation of the sample [3]. SPE separation is based on the selective distribution of analytes between the solid packing material and liquid mobile phase. Generally, SPE consists of four steps: column prewashing (conditioning), sample loading, column postwashing, and sample desorption [2]. The relationships between the target compounds and the sorption, centered on the solid phase, include hydrophobic interactions such as van der Waals forces and hydrophilic interactions such as dipole-dipole, hydrogen bonding, and π–π interactions [2].

SPE is carried out using either silica-based or polymer-based sorbents. Silica-based sorbents are mainly used in the case of nonpolar to midpolar analytes. The silica-based sorbents are modified with groups such as C8, C18, Ph, or CN. The commonly used C18 sorbents and their modifications are applied in biomedicine, pharmacology, and toxicology for extraction from different biological matrices. Polymer-based sorbents (such as PGC, XAD, PDMA) have different advantages, including no need for acidic/basic elution modifiers, no pH limitations, and a higher loading capacity. Porous Graphitic Carbon (PGC) is commonly used. PGC SPE cartridges offer retention and separation of polar compounds [4].

There is no doubt that the market for SPE cartridges is increasing. However, the scale-up and industrial application of SPE has been limited [5]. Starbon is a family of renewable, carbonaceous, and mesoporous materials with tunable surface functionality and structures [6]. These materials are derived from abundant and inexpensive residual polysaccharides, such as starch, alginic acid, or pectin [1]. The first member of this family of porous carbon-based materials prepared from starch was defined under the patented and trademarked name “Starbon” [6]. Production exploits the natural tendency of polysaccharide gels to form nano-channel biopolymer structures. These carbon-based materials are highly porous and mechanically stable in the temperature preparation range from 200 to 1000 °C [7]. The surface functionality (hydrophilicity or hydrophobicity properties) of Starbon can be adjusted by changing the carbonization temperature [7]. As a patented and biomass-based material, the surface chemistry of the Starbon range from 200 to 1000 °C has already been characterized by different analytical techniques such as X-Ray photoelectron spectroscopy (XPS), thermogravimetric, 13C MAS NMR and DRIFT, FTIR spectroscopy, and SEM [7].

Starbon is produced from bio-based materials such as starch, alginic acid, and pectin. A green and sustainable approach was used to make the Starbon A800 used in this paper. Starbon A800 is produced from alginic acid at a carbonization temperature of 800 °C. In other applications, it has been found to be excellent for purification purposes as it strongly or permanently retains analytes [8]. The fullerene-like surfaces promote adsorption by allowing specific affinities for both planar and nonplanar molecules. Starbon A800 has a more heterogeneous, fine, fibrous structure. It has been shown to convert into aromatic 2D graphite-like systems. Its mechanism of adsorption is based mainly on the hydrophilicity or hydrophobicity properties of its pores [8].

In this study, Starbon A800 was used as the adsorbent for SPE and compared with currently commercially used C18 and PGC adsorbents. Conditions were first optimized for Starbon A800. The extraction capabilities of Starbon^®^ A800, C18, and PGC were investigated and compared for 25 compounds of various types and polarities using different types of eluting solvents. Finally, Starbon A800 SPE was applied to real samples (wine and tap-water).

This is the first extensive study on SPE optimization and applications using Starbon A800. Starbon A800 is a promising, low-cost, green, scalable, alternative adsorbent for use in the SPE market for the extraction of many organic compounds over a wide range of polarities.

## 2. Materials and methods

### 2.1. Chemicals and materials

Alginic acid, HPLC grade solvents (methanol, ethanol, dichloromethane, heptane), *N*-nitrosodimethylamine (NDMA), *N*-nitrosodiethylamine (NDEA), *N*-nitrosopyrrolidine (NPYR), *N*-nitrosomorpholine (NMOR), *N*-nitrosodi-n-propylamine (NDPA), *N*-nitrosopiperidine (NPIP), *N*-nitrosodi-n-butylamine (NDBA), carvone, linalool, linalyl acetate, syringol, resorcinol, guaiacol, vanillin, propanil, simazine, imazalil, acetochlor, chloropyrifos, thiobendazole, naphthalene, 1-naphthol, 1-napthylamine, aniline, imidazole, caffeine, phenethyl alcohol, furfural, methyl-2-furoate, and decanoic acid were purchased from Merck (Gillingham, Dorset, UK). These standards were dissolved in methanol at a concentration of 5 mg**/**mL of each nitrosamine. Working solutions were then prepared by an appropriate dilution of this stock solution with methanol. Stock solutions were protected from light and stored at 4 °C. Sodium hydroxide and hydrochloric acid were obtained from Thermo Fisher Scientific (UK).

C18 SPE cartridges (100 mg; 1 mL tube) were purchased from Merck (Gillingham, Dorset, UK). PGC SPE cartridges (100 mg; 1 mL) were obtained from Thermo Fisher Scientific (UK).

### 2.2. Characterization of materials

A Micromeritics ASAP2020 volumetric adsorption analyser was employed to evaluate the porosity of the fabricated Starbon A800. The Brunauer-Emmett-Teller (BET) equation was used to calculate the specific surface area (S BET). The mean pore diameter (dp) was determined using the Barrett-Joyner-Halenda (BJH) model for the full range of mesopores [9].

### 2.3. Preparation of Starbon A800

Alginic acid (40 g) was combined with deionized water (800 mL) and heated at 90 °C for 2 h. The water in the gel was replaced twice with ethanol (2 × 800 mL) which is a solvent with lower-surface tension. Drying was performed using supercritical CO_2_ at 40 °C, 100 bar, for 3 h. The resulting material was heated in an inert atmosphere at 1 °C/min to the required temperature for carbonization; in this case, 800 °C for Starbon A800. The full method for the preparation of Starbon A800 is detailed in Budarin et al. [6].

### 2.4. Optimisation of Starbon A800

An optimization study, using the 7- nitrosamine mixture as a standard, was carried out to optimise the potential parameters which may affect the performance of Starbon A800 SPE. This included different elution solvents, different solvent volumes, flow rate, pH value, sample volume, amount of adsorbent, and the selection of drying method.

### 2.5. Solid phase extraction procedure

The empty SPE cartridges were packed with 100 mg of Starbon A800. To hold the packed Starbon A800 in place, the polypropylene upper and lower frits were kept in place at each end of the cartridge. A SPE automated vacuum manifold (24-port model) was used (Supelco, Gillingham, Dorset, UK). SPE cartridges (Starbon A800, C18, and PGC) were conditioned using dichloromethane (10 mL DCM) followed by methanol (10 mL) and deionized water (10 mL). A known volume of sample was then applied to the cartridge. Following that, the retained compounds were eluted using an optimum volume of the selected solvent and lastly, the extract was dried. The final volume of the sample was 0.5 mL. The same procedure was followed with other elution solvents (heptane and ethanol).

### 2.6. Real sample extraction

Samples of French merlot red wine (from a local supermarket) and tap water (from the Green Chemistry research lab) were directly processed without any pretreatment. The previously optimised SPE conditions were used for both samples. The Starbon A800 SPE cartridges (100 mg) were first conditioned with DCM (10 mL), followed by methanol (10 mL) and deionized water (10 mL). Then 100 mL of red wine or 300 mL of tap water was added to the cartridges. Retained chemicals were eluted using 13 mL of DCM. Eluted samples were dried to a final volume of 0.5 mL using N_2_ blowing and, following this, anhydrous sodium sulphate was added to absorb any remaining water. The results are the mean of four experiments and the relative standard deviation was in the range of 2.6%–11.3%.

At this point, the extraction stages of the study had been completed and the Starbon A800 was disposed of safely in the laboratory furnace by burning at high temperature. The resulting residue is considered nontoxic and can be safely placed in normal waste.

### 2.7. Nitrosamine mixture analysis

Analysis for the 7-nitrosamine mixture SPE optimisation study was carried out using two-dimensional gas chromatography with a nitrogen chemiluminescence detector (GC xGC-NCD) system. The GCxGC-NCD system consisted of an Agilent 7890 gas chromatograph and an Agilent 255 Nitrogen chemiluminescence detector (Agilent Technologies, Palo Alto, CA, USA). The modulator between the first and second GC columns was based on a Leco (Cheshire, UK) liquid nitrogen two-stage cold jet system. The first column was a nonpolar BPX5 (30 m × 0.32 mm i.d. × 0.25 μm film thickness) and the second column was a BPX50 (1.5 m × 0.10 mm i.d. × 0.10 μm film thickness). Both were obtained from SGE Analytical Science (VIC, Australia). Over the entire course of the analysis, data from the NCD was collected at 50 Hz. The modulator secondary oven was operated at +15 °C above the GC oven temperature. The first-dimension separation axis went up to 840 s and the second-dimension axis ended at 5 s. The carrier gas was helium. The initial temperature of the first-dimension column was 60 °C for 1 min and the subsequent temperature programme was a heating rate of 10 °C/min until 180 °C was reached and held isothermally for a further 1 min. The initial temperature of the second-dimension column was 75 °C for 1 min and a 10 °C/min heating rate was employed until 195 °C was reached and held isothermally for a further 1 min. Individual standards were used for peak identification. The sample (1 μL) was directly injected using a splitless method into the GCxGC-NCD using a Gerstel automated liquid injector (Gerstel, Mulheim an der Ruhr, Germany).

### 2.8. Red wine and tap water analysis

A two-dimensional gas chromatography with time of flight/mass spectrometry (GCxGC-TOF/MS) system was used to analyze the eluted red wine and tap water samples. It consisted of an Agilent 6890 (Agilent Technologies, Palo Alto, CA, USA) gas chromatograph and a Pegasus III TOF-MS (LECO, St. Joseph, MI, USA). The modulator between the first and second GC columns was based on a Leco (Cheshire, UK) liquid nitrogen two-stage cold jet system. The carrier gas was helium, used at a constant flow of 1.0 mL/min. One μL of sample volume was injected using the splitless method. The same column combination was used here as with the GCxGC-NCD system described above. The initial temperature of the first-dimension column was 60 °C for 1 min and the subsequent temperature programme was a heating rate of 10 °C/min until 300 °C was reached and held isothermally for a further 5 min. The initial temperature of the second-dimension column was 75 °C for 1 min and a 10 °C/min heating rate was used until 315 °C was reached and held isothermally for a further 5 min. TOF/MS with electron ionisation was used for peak identification. The mass spectrometer used a push plate frequency of 5 kHz, with transient spectra averaging to give unit resolved mass spectra between 45 and 350 u at a rate of 50 spectra s-1. Peaks in the total ion chromatogram (TIC) profiles for the compounds were characterized or tentatively identified from their mass spectral data using the NIST and Wiley mass spectrometry libraries.

#### 2.8.1. UV-visible spectroscopy

Twenty-five compounds were analyzed by the UV-vis spectrometer to compare SPE capability using either Starbon A800, C18, or PGC adsorbents. The calibration curves of each analyte were plotted through five points: 1, 5, 10, 18, and 25 ppm. Then the spectra were measured under the scanning mode of Jasco V-550 UV-vis, the highest peak and corresponding identifying UV wavelengths were determined based on each spectrum. Based on each calibration graph, the linearity coefficient (R^2^) and the coefficient equation were calculated, and the measured UV-absorbance intensity of each sample could be converted into the concentration of each selected compound. Quantitative analysis of each compound from the solid phase extraction (Starbon A800, C18, and PGC) was calculated based on the slopes of the calibration curves. The results are the mean of the three experiments (N = 3).

## 3. Results and discussion

### 3.1. Physical properties of Starbon A800

Starbon A800 was prepared according to the patented method [6] to begin to investigate the possibility of it being used as an adsorbent in solid-phase extraction. Two different batches were used in this study as one was found to be not enough. In Table 1, the characterization data of the two prepared batches can be found; both showed similar physical parameters. The definition of a mesoporous material is one which contains pores of between 2–50 nm. Mesoporous materials have attracted significant attention, such as in the separation and adsorption of organic molecules [7]. Mesoporous carbons have advantages over classical microporous materials such as chemical inertness and stability. Different pore dimensions result in diversity in adsorption properties which give rise to their varied already investigated and potential future applications. System filtering properties are significantly aided by the presence of macropores, and this also enhances further the flow/mass transfer/diffusion properties of the material.

C18 Cartridges feature a highly retentive alkyl-bonded phase for nonpolar to midpolar compounds. The hydrophobic reversed phase material is retentive for most nonpolar compounds and retains most organic analytes from aqueous matrices. C18 has mesoporosity (5 nm), a large surface area (654 m^2^/g), and a small total pore volume (0.71 cm^3^/g) [10]. PGC materials possess excellent textural properties, which make them ideal stationary phase media for chromatography and the extraction of midpolar to polar compounds. PGC has high mesoporosity (35 nm), low surface area (120 m^2^/g), and a large total pore volume (0.85 cm^3^ /g) [7]. Starbon A 800 also has mesoporosity (average 19 nm), and a surface area (average 439 m^2^/g) but has the largest total pore volume compared with C18 and PGC (1.44 cm^3^/g). Changing the carbonization temperature during preparation of Starbon materials alters pore size and it has been seen that increasing the carbonization temperature from 0–1000 °C during preparation decreased the total pore volume from 2.7 cm^3^/g to 1.1 cm^3^/g [8].

### 3.2. Optimisation of Starbon A800 solid phase extraction parameters using a 7-nitrosamine standard mixture

Nitrosamines are popular compounds. Seven nitrosamines were selected for use in this part of the study to optimize the SPE capability of Starbon A800. This study followed the method used by Liao et al. [11] and Jurado-Sanchez et al. [12] when they extracted nitrosamines from water samples using C18 as their adsorbent. The same seven nitrosamines (NDMA, NDEA, NPYR, NMOR, NDPA, NPIP, and NDBA) were extracted in this study; however, Starbon A800 was used as the adsorbent. The results from using the Starbon A800 were comparable with those from both the above references.

There are many factors that may influence the enrichment efficiencies of the nitrosamine compounds. To determine the optimum factors that ensure the entire recovery of all the analytes adsorbed, the effects of the type of elution solvent and its volume, the sample flow rate, the sample pH, the sample volume, and the mass of adsorbent were investigated.

To ensure the complete elution of the target nitrosamine from the cartridge, a solvent of optimum polarity should be chosen. For this purpose, the following three organic solvents with different polarities were tested: hexane, dichloromethane (DCM), and ethanol. Elution of nitrosamine from the cartridge was performed using 10 mL of the selected solvent. It seems clear that the recovery of nitrosamine compounds is related to the solvent polarity. This is due to different partition constants of nitrosamine between the Starbon A800 (solid phase) and solvent (liquid phase). Casado et al. [10] used methanol and acetonitrile as elution solvents for extracting drug residues and Bruzzaniti et al. [13] used methanol and acetone as elution solvents for extracting herbicides from drinking water. Both publications [10,13] demonstrated similar percentage recoveries of selected chemicals using different SPE cartridges and various types and amounts of elution solvents. One of the main parameters affecting the recovery of analytes is solvent polarity. The experimental results demonstrated that DCM, with a medium polarity, gave a much higher elution efficiency than hexane and ethanol for all the target nitrosamines, therefore DCM was selected as the elution solvent for the next optimisation steps.

**Figure 1:**
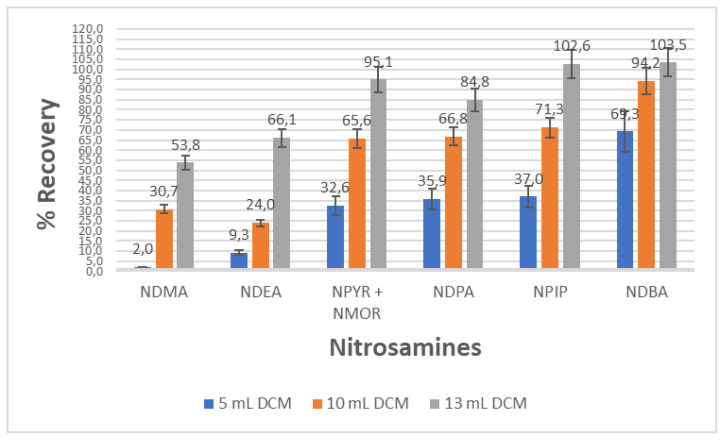
Influence of volume of the solvent on the adsorption and desorption of 7 nitrosamines using Starbon A800 (The results are the mean of three experiments).

The volume of the solvent has a great effect on the elution performance and efficiency. To determine the required DCM volume to recover all the analytes from the Starbon A800 packed cartridge, solvent volumes in the range of 5–13 mL were tested. From the results displayed in Figure 1, it appeared that the nitrosamine compounds needed a volume of solvent 13 mL to get the best recoveries. It should be noted, in Figure 1, that NPYR and NMOR appeared as coelutes in the chromatograms. It was not possible to separate them further so both compound values are given together.

The influence of sample flow rate was investigated over a range of 1–4 mL**/**min with the other conditions kept constant. Flow rate changes did not make a great difference to the results. The experimental results meant that the lowest flow rate (1 mL/min) was selected for use in further experiments.

The effect of sample pH on the recoveries of the selected nitrosamines was examined over a range of 4–9. The most satisfactory recoveries were obtained at pH 6,7, and 8.

**Figure 2:**
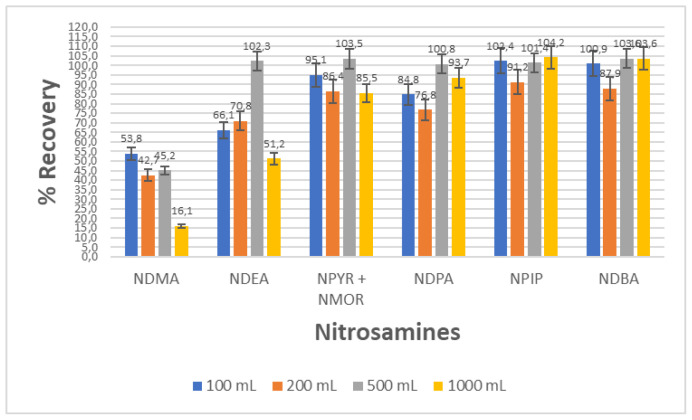
Effect of sample volume on adsorption and desorption of 7 nitrosamines using Starbon A800 (The results are the mean of three experiments).

The sample volume used in the solid phase extraction was investigated. Different volumes (100, 200, 500, and 1000 mL) of distilled water were spiked with 0.1 μg**/**mL of each analyte and then preconcentrated using Starbon A800 packed cartridges. As shown in Figure 2, overall recovery levels obtained for the studied nitrosamines were good. However, NDMA and NDEA recovery significantly decreased when the water volume was increased to 1000 mL.

The effect of the amount of Starbon A800 packed into the solid-phase column on the recovery of analytes was studied. Distilled water (100 mL) was spiked with 0.1 μg/mL of each analyte and then preconcentrated using Starbon A800 packed cartridges. Three amounts (100, 200, and 500 mg) were tested for their efficiency in the preconcentration of nitrosamine compounds in spiked distilled water. The results obtained showed insignificant contrasts in recoveries among the different amounts of cartridge packing. Therefore, an amount of 100 mg of Starbon A800 was used for further SPE experiments.

The selection of a suitable drying method after loading the sample plays an important role in the SPE procedure and affects the extraction efficiency. Three drying methods were investigated in this paper: the first one was using nitrogen to blow the sample until it was visibly dry. The second was using nitrogen blowing until the sample volume was approximately 0.5 mL and then adding anhydrous sodium sulphate to absorb any remaining water. The third method was using a vacuum pump for 30 min until the sample was visibly dry. It was found that a significant loss of nitrosamine compounds occurred if the sample was left wet, however, the same loss also occurred if the sample was fully mechanically dried (using solely blowing or vacuum) because the nitrosamine compounds evaporated under such conditions. Therefore, the second method was used as the drying step.

Parameters investigated in the optimization of Starbon A800 solid phase extraction (elution solvent, sample flow rate, pH, amount of adsorbent, and drying method) using a 7-nitrosamine standard mixture are shown in Supplementary Table 1. The optimization results are the mean of three experiments and the relative standard deviation values are given in this Table.

### 3.3. Separation of various types of compounds using Starbon A800, C18, and PGC

Diversity in the adsorption properties of different materials results in their varied investigated, current, and potential future applications. Micropores are ideally suited to liquid and gas-phase adsorption due to strong van-der-Waals interactions. In contrast, mesopores are more suitable for chromatographic separation, as these pore sizes allow a high loading of accessible active sites and, importantly, provide efficient diffusion/mass transfer of liquid phase analyte or substrate [14]. C18 has a stronger affinity for hydrophobic or less polar compounds, whereas PGC is an important tool in the separation of polar compounds [13]. Table 2 shows a comparison of SPE using Starbon A800, C18, and PGC with various types of polar and nonpolar chemicals such as terpenes, phenolics, pesticides, PAHs, amines & nitrosamines. Linearities of the selected 6 various group compounds and DCM and ethanol elution solvent data have been given in Supplementary Table 2.

Bielicka-Daszkiewicz [15] used Hansen solubility parameters for the selection of the most favourable analyte/sorbent/solvent system for SPE. A sorbent–solvent system comprised of a selection of eight polymeric sorbents and seven solvents used as eluents was employed for the extraction of phenol and its oxidation products [16]. Hansen solubility parameters can be used to aid solvent selection for the extraction or elution of a desired target molecule [17].

Solvents can dissolve, dilute, or extract other compounds without chemically altering them. In this study, solvents with different polarities: namely, ethanol, DCM, and heptane were tested as elution solvents. Twenty-five compounds with different chemical characteristics were selected to compare their SPE capability using Starbon A800, C18, and PGC adsorbents. Hansen solubility parameters (δ_d_, δ_p_, δ_h_) of the elution solvents and the 25 compounds were found within the literature [18] and are displayed in Table 3. Solubility is defined as the degree to which a substance dissolves in a solvent to make a solution. Extraction relies on differences in solubility, expressed as the distribution coefficient (the ratio of a material’s solubilities in two solvents). Therefore, solubility data can be used to choose an appropriate solvent for an extraction. The Hansen solubility parameters of any compound can aid in the selection of an appropriate adsorbent and elution solvent. Tools are needed for the fast assessment of solubilizing properties to widen the range of applications of solvents particularly emerging sustainable solvents.

The results in Table 2 show that PGC performed slightly better with terpenes, phenolics, and pesticides. As expected, C-18 worked better with polycyclic aromatic hydrocarbons (PAHs). Starbon A800 performed best with amines and nitrosamines and compounds classed as ‘other’ in Table 2. Overall, DCM was the better elution solvent compared with ethanol and heptane. Heptane performed well especially with nonpolar compounds, however, overall, ethanol and DCM performed more favourably. Using SPE columns packed with 100 mg of Starbon A800, the overall recovery of the 25 compounds was the best (82.6%) when DCM was used as the elution solvent. This is an encouraging result for SPE applications on the recovery of various types of compounds over a differing range of polarities. The best average SPE recoveries found for the 25 compounds of varying polarity, using DCM as the elution solvent, were 83, 79, and 65% using Starbon A800, PGC, and C18 respectively.

Starbon A800, C18, and PGC are all classed as mesoporous materials. Of them, C18 has the smallest diameter pores, but the biggest surface area. PGC has the largest diameter pores, but the smallest surface area. Starbon A800 falls somewhere between C18 and PGC for both parameters. So, when compared with C18 and PGC, although Starbon A800 may have only medium-sized mesopores, it still has a relatively high surface area, meaning that when the total pore volume is calculated, it has the largest total pore volume out of C18 and PGC. This quality of falling between C18 and PGC, yet still having the largest total pore volume may explain why Starbon A800 is able to adsorb a greater spectrum of varying polarity compounds than either C18 or PGC.

The same Starbon A800 material was used 5 times in a row with the 6 different types of compounds (linalool, guaiacol, simazine, 1-napthyamine, N-nitrosopyrrolidine, and phenethyl alcohol) with two elution solvents (ethanol and DCM). As can be seen from Supplementary Table 3, data was consistent with RSDs between 2.8%–6.7%. This clearly demonstrates Starbon A800’s reusability with consistent adsorption capability for these 6 different types on compounds and 2 elution solvents.

Reproducibility was tested using two batches of Starbon A800. The two different batches showed similar physical parameters as seen in Table 1. BET Surface area, total pore volume, and BJH desorption diameter did not differ much between both batches. Both Starbon A800 batches were used during this work and did not show any significant difference in reproducibility.

### 3.4. Starbon A800 SPE application to real samples

There has been much work on the use of various SPE adsorbents in different real sample applications. A hydrophilic hyper crosslinked polymer based on natural kaempferol showed highly effective extraction of 5-nitroimidazoles in environmental water, honey, and fish samples [19]. A magnetic o-hydroxyazobenzene porous organic polymer was applied to extract phthalate esters (PAEs) from plastic bottled juice [20]. In this study, following the optimized extraction procedures, red wine and tap water samples were used as ‘real samples.’

Wine volatiles are commonly analyzed using GC-MS. Sample preparation before GC analysis is essential. Common sample preparation techniques are liquid-liquid extraction (LLE) [21], solid phase extraction (SPE) [22], solid phase microextraction (SPME) [23], and stir bar sorptive extraction (SBSE) [24]. Comprehensive two- dimensional gas chromatography (GC×GC) is a powerful technique for the analysis of complex volatile fractions of wine [25]. GC×GC allows the separation of a large number of compounds in a single chromatographic run due to the added selectivity of the second column [25]. The combination of GC×GC with time-of-flight mass spectrometry (TOF/MS) adds an extra dimension of information in terms of full mass spectral data acquisition and mass spectral continuity, which permits the deconvolution of spectra for coeluting peaks [25].

The Starbon A800 SPE method was applied to extract red wine volatiles. The total number of components extracted and analysed was 27 (Table 4). The results are the mean of four experiments and the relative standard deviation was in a range of 2.6%–11.3%. The major components were phenylethyl alcohol, levoglucosan, 4-ethylphenyl acetate, ethyl hydrogen succinate, and butanedioic acid diethyl ester. Among all the chemical groups found in the volatile content of French Merlot wine, phenolics were present in the highest number (7), followed by acids (6), alcohols (5), esters (3), and amines (2). As can be seen from Table 4, there are some components which can only be separated on the second column such as octanoic acid and ethyl hydrogen succinate. Similar volatiles have previously been extracted from wines [26]. Colour does not affect SPE extraction from red wine [22,26]. Red wine samples do not need filtration before SPE procedures. GCxGC-TOF/MS chromatogram of the red wine sample is shown in Figure S1.

A 300 mL tap water sample was passed through the Starbon A800 packed SPE cartridge. 0.5 mL concentrated extract was analyzed using GC×GC with TOF/MS. GC×GC with TOF/MS enabled the identification of 69 compounds in tap water. Table 5 is a list of them, with their retention times and relative abundances. Three of the standard compounds from Table 3 (resorcinol, phenylethyl alcohol, and decanoic acid) are seen in Table 5. The main groups found were 13 aldehydes, 11 alcohols, 8 ketones, 8 hydrocarbons, 6 acids, 3 esters, 2 amines, 2 phenols, 1 nitrosamine and 1 PAH. The major compounds were 1-methyl-2-pyrrolidinone (17.94%), 2-methyl-2-propanol (9.38%), heptacosane (6.19%), 2-propen-1-ol (4.39%) and acetophenone (4.29%). Many studies have been performed to identify organic compounds from drinking water using SPE systems. Jurado-Sanchez et al. [12] extracted 17 amines and 10 nitrosamines from water samples using C18 as their adsorbent. Bruzzoniti et al. [13] determined 13 pesticides/herbicides from drinking water. GCxGC-TOF/MS chromatogram of the tap water sample is shown in Figure S2.

Starbon A800 SPE adsorbent was seen to successfully extract various types of compounds from wine and tap water.

## 4. Conclusions

In this study, a simple method was set up to determine 25 compounds using Starbon A800 as a novel adsorbent in an SPE system and compare it with currently used adsorbents, C18 and PGC. The overall best recovery of the 25 compounds (82.6%) was achieved using 100 mg of Starbon A800 with DCM as the elution solvent. The optimised Starbon A800 SPE method was applied successfully to red wine and tap water samples. Twenty-seven compounds were found in red wine and 69 compounds from tap water. C18 is normally used as an adsorbent in SPE for nonpolar to mid-polar compounds, whilst PGC is used for mid-polar to polar compounds. However, Starbon A800 has been seen to perform well across the whole range of polarities. It is thought this is due to Starbon A800 having a larger total pore volume than C18 and PGC. Pilot scale, cost-effective Starbon A800 was produced. However, this still costs more than C18 and PGC. Further work needs to be carried out to find out if there is a market for this material as then the decreasing cost may enable it to be more competitive with the existing SPE materials.

## Supplementary Information

**Table S1:** Optimization of Starbon A800 solid phase extraction parameters (elution solvent, sample flow rate, pH, amount of adsorbent, and drying method) using a 7-nitrosamine standard mixture.

Optimization Condition	Nitrosamine percentage recoveries					

	NDMA	NDEA	NPYR + NMOR	NDPA	NPIP	NDBA
**Elution Solvent**						
DCM	30.7 (8.8)	24.0 (8.7)	65.6 (8.1)	66.8 (4.6)	71.3 (4.9)	94.2 (7.0)
Ethanol	28.6 (3.9)	26.2 (4.7)	69.5 (5.3)	63.2 (3.7)	68.9 (5.0)	90.5 (6.6)
Hexane	25.7 (8.1)	22.1 (7.7)	59.0 (4.8)	60.9 (3.9)	55.8 (7.2)	80.0 (6.8)
**Sample Flow rate (mL/min)**						
1	53.8 (5.4)	66.1 (7.3)	95.1 (6.5)	84.8 (8.5)	102.6 (5.6)	103.5 (6.9)
2	59.2 (4.2)	64.1 (5.7)	88.6 (7.1)	85.0 (6.2)	99.1 (8.5)	98.6 (7.2)
3	55.1 (3.8)	62.1 (8.5)	90.3 (6.3)	88.8 (7.6)	100.9 (3.8)	95.6 (6.7)
4	57.4 (7.7)	69.1 (5.6)	93.7 (6.4)	80.6 (7.1)	88.9 (4.6)	93.5 (8.0)
**pH**						
4	15.2 (8.8)	24.6 (9.1)	21.4 (5.9)	32.2 (4.8)	25.0 (6.6)	54.1 (5.1)
5	43.2 (4.4)	55.5 (6.2)	88.3 (3.8)	78.6 (6.1)	80.8 (4.8)	88.6 (4.5)
6	55.7 (3.9)	63.8 (7.8)	99.0 (8.6)	88.3 (2.8)	96.7 (6.6)	101.4 (8.7)
7	53.8 (5.4)	66.1 (7.3)	95.1 (6.5)	84.8 (8.5)	102.6 (5.6)	103.5 (6.9)
8	57.8 (6.7)	62.3 (4.7)	91.9 (4.6)	86.9 (6.1)	100.5 (7.3)	98.9 (4.4)
9	43.6 (3.9)	55 (6.7)	87.3 (5.6)	76.9 (3.3)	99.3 (6.4)	98.2 3.7)
**Amount of Starbon A800 (mg)**						
100	53.8 (5.4)	66.1 (7.3)	95.1 (6.5)	84.8 (8.5)	102.6 (5.6)	103.5 (6.9)
200	55.9 (4.7)	63.8 (5.5)	92.1 (3.0)	93.6 (8.2)	99.9 (6.9)	100.8 (5.5)
500	50.6 (2.9)	59.2 (6.4)	80.8 (4.8)	95.7 (7.6)	103.6 (6.1)	104.1 (6.0)
**Drying method**						
Nitrogen blow until dry	22.1 (9.1)	45.9 (7.7)	56.9 (5.7)	88.4 (3.6)	95.5 (5.6)	98.2 (6.6)
Nitrogen blow until 0.5mL	53.8 (5.4)	66.1 (7.3)	95.1 (6.5)	84.8 (8.5)	102.6 (5.6)	103.5 (6.9)
Vacuum pump until dry	46.6 (7.2)	55.8 (6.6)	93.6 (4.8)	89.2 (2.8)	96.3 (4.8)	99.0 (7.3)

As identified by GCxGC-NCD. The results are the mean of three experiments and the data mentioned in parentheses are the corresponding relative standard deviations.

**Table S2:** UV Linear range of selected compounds.

Compound	Elution solvent	Linear range (ppm)	Calibration curve formula	Coefficient of determination
Carvone	DCM	2–25	y = 0.065x + 0.0074	R ^2^ = 0.9997
Carvone	Hexane	2–25	y = 0.0698x + 0.0011	R ^2^ = 0.9996
Resorcinol	DCM	5–100	y = 0.0123x	R ^2^ = 0.99927
Resorcinol	Hexane	5–100	y = 0.0139x	R ^2^ = 0.99742
Propanil	DCM	1–20	y = 0.0828x + 0.0419	R ^2^ = 0.9996
Propanil	Hexane	1–20	y = 0.0901x–0.0391	R ^2^ = 0.9994
Naphthalene	DCM	0.5–5	y = 0.2941x + 0.0221	R ^2^ = 0.9998
Naphthalene	Hexane	0.5–5	y = 0.1193x–0.0453	R ^2^ = 0.9958
Methyl-2-furoate	DCM	1–25	y = 0.0953x + 0.0318	R ^2^ = 0.9994
Methyl-2-furoate	Hexane	1–20	y = 0.1005x + 0.0326	R ^2^ = 0.9997
NNPYR	DCM	1–20	y = 0.048x + 0.1383	R ^2^ = 0.9990
NNPYR	Hexane	1–20	y = 0.052x–0.0303	R ^2^ = 0.9994

**Table S3:** Reusability of Starbon A800 adsorbent for selected compounds and elution solvents.

Compound (elution solvent)	% Recovery from reuse of Starbon A800 adsorbent					Average	SD	RSD

	1 ^st^ time	2 ^nd^ time	3 ^rd^ time	4 ^th^ time	5 ^th^ time			
Phenethyl alcohol (EtOH)	85.2	89.6	84.2	88.6	91.0	87.7	2.9	3.3
Phenethyl alcohol (DCM)	97.0	102.2	95.1	98.2	100.4	98.6	2.8	2.8
1-Naphthylamine (EtOH)	51.8	61.5	55.9	56.0	53.1	55.7	3.7	6.7
1-Naphthylamine (DCM)	83.7	77.9	85.0	82.9	78.9	81.7	3.1	3.8
Linalool (EtOH)	104.6	93.5	99.6	93.5	97.3	97.7	4.7	4.8
Linalool (DCM)	102.1	98.2	96.3	98.9	95.7	98.2	2.5	2.6
Simazine (EtOH)	102.9	99.8	92.5	94.6	101.2	98.2	4.4	4.5
Simazine (DCM)	104.7	101.3	97.8	96.5	103.6	100.8	3.6	3.5
Guaiacol (EtOH)	81.3	78.9	85.1	88.3	78.6	82.4	4.2	5.1
Guaiacol (DCM)	103.5	100.5	96.3	98.8	102.1	100.2	2.8	2.8
N-nitrosopyrrolidine (EtOH)	105.1	103.2	95.9	97.7	99.4	100.3	3.8	3.8
N-nitrosopyrrolidine (DCM)	102.7	95.1	98.2	93.9	100.6	98.1	3.7	3.7

SD: Standard deviations, RSD: Relative standard deviations

Figure S1 GCxGC-TOF/MS chromatogram of red wine after Starbon A800 extraction.

Figure S2 GCxGC-TOF/MS chromatogram of tap water after Starbon A800 extraction.

## Figures and Tables

**Table 1:** Porosimetry data for batches of prepared Starbon A800, C18, and PGC.

Material	Starbon A800			C18 (Casado et al., 2017)	PGC (White et al., 2009)
	Batch 1	Batch 2	Average		
BET surface area (m^2^/g)	443	435	439	654	120
Total pore volume (cm^3^/g)	1.46	1.42	1.44	0.71	0.85
BJH desorption diameter (nm)	19.2	18.8	19.0	5.0	35.0

**Table 2:** SPE applications of Starbon A800, C18, and PGC on various types of compounds. The results are the mean of the three experiments and the relative standard deviation was in the range of 1.2%–10.5%.

	γ_max_ (nm)	Starbon^®^ A800			C18			PGC		

		Ethanol	DCM	Heptane	Ethanol	DCM	Heptane	Ethanol	DCM	Heptane
**Terpenes**										
Carvone	280	81.0	101.3	76.8	103.4	105.2	26.7	105.0	102.7	106.1
Linalool	320	104.6	102.1	65.3	102.8	89.4	56.2	104.2	107.7	76.3
Linalyl acetate	612	94.1	55.5	45.5	83.8	67.9	32.3	66.5	41.5	33.2
**Phenolics**										
Syringol	206	57.8	100.8	0.0	106.3	96.6	6.8	105.3	102.9	106.8
Resorcinol	273	53.3	68.2	0.0	0.0	16.7	0.0	68.4	87.3	28.4
Guaiacol	274	81.3	103.5	41.7	70.5	55.2	69.2	107.9	105.5	101.6
Vanillin	372	66.7	47.5	0.7	53.2	46.9	2.7	98.6	107.0	45.0
**Pesticides**										
Propanil	258	2.4	88.4	65.8	100.9	103.7	25.2	103.8	100.9	46.8
Simazin	223	102.9	104.7	98.2	95.5	106.2	90.7	93.6	104.4	88.9
Imazalil	230	27.9	70.3	0.6	95.4	17.2	0.6	91.3	98.8	58.7
Acetochlor	243	36.1	64.4	61.5	52.6	61.5	84.0	50.4	79.8	102.7
Chloropyrifos	290	30.3	102.6	59.0	30.6	76.6	42.5	53.7	94.4	108.6
Thiobendazole	298	48.7	17.9	0.2	83.1	51.2	0.2	80.3	98.7	0.2
**PAHs**										
Naphthalene	222	51.8	83.7	14.1	76.1	89.4	86.6	65.8	68.9	95.0
1-naphthol	241	70.5	64.9	0.0	26.4	103.7	107.0	82.2	61.8	103.5
1-napthylamine	548	57.7	90.4	0.5	99.0	104.3	65.7	106.7	103.3	97.3
**Amines and nitrosamines**										
Aniline	230	49.6	104.2	8.1	11.1	31.1	12.6	25.7	26.8	18.6
Imidazole	415	55.9	38.4	76.8	28.1	36.4	58.8	10.3	20.0	44.3
N-nitrosopyrrolidine	242	105.1	102.7	101.6	27.2	35.8	104.8	22.4	68.8	108.2
N-nitrosodi-n-butylamine	240	103.8	106.0	103.9	100.2	102.2	57.5	105.1	106.3	104.4
**Others**										
Caffeine	271	105.3	102.0	94.4	44.5	31.1	60.2	88.6	103.5	64.9
Phenethyl alcohol	340	85.2	97.0	14.2	92.6	71.0	42.7	90.9	74.2	88.1
Furfural	278	70.2	86.4	13.3	16.7	23.4	0.0	21.1	11.2	52.5
Methyl-2-furoate	230	9.1	91.7	76.3	19.4	30.9	40.5	12.2	49.5	65.2
Decanoic acid	205	1.5	107.0	79.5	12.2	103.3	55.7	2.0	107.8	68.4

**Average recovery**		61.2	82.6	43.7	60.7	65.1	44.7	69.0	79.3	70.9

**Table 3:** Hansen solubility parameters for elution solvents and selected compounds.

Compounds	m/z	CAS	Hansen solubility parameters		

			_δ_d	_δ_p	δ_h_
**Elution solvents**					
Ethanol	46	64–17–5	15.8	8.8	19.4
DCM	85	75–09–2	17.0	7.3	7.1
Heptane	100	142–82–5	15.3	0.0	0.0
**Terpenes**					
Carvone	150	6485–40–1	17.5	5.7	3.8
Linalool	154	126–91–0	16.7	2.8	6.7
Linalyl acetate	196	115–95–7	16.3	2.0	3.5
**Phenolics**					
Syringol	154	91–10–1	19.3	7.6	13.7
Resorcinol	110	108–46–3	18.6	8.1	20.3
Guaiacol	124	90–05–1	18.0	7.0	12.0
Vanillin	152	121–33–5	19.4	9.8	11.2
**Pesticides**					
Propanil	218	709–98–8	19.9	11.3	7.1
Simazin	202	122–34–9	19.5	12.1	9
Imazalil	297	35,554–44–0	19.8	6.4	3.6
Acetochlor	270	34,256–82–1	18.5	9.6	5.0
Chloropyrifos	351	2921–88–2	18.6	11.6	6.0
Thiobendazole	201	148–79–8	21.6	9.3	8.2
**PAHs**					
Naphthalene	128	91–20–3	19.2	2.0	5.9
1-naphthol	144	90–15–3	19.7	6.3	12.3
1-napthylamine	143	134–32–7	20.4	5.1	9.1
**Amines and nitrosamines**					
Aniline	93	62–53–3	20.1	5.8	11.2
Imidazole	68	288–32–4	19.6	12.4	11.5
N-nitrosopyrrolidine	100	930–55–2	17.3	8.2	6.9
N-nitrosodi-n-butylamine	158	924–16–3	15.7	4.7	3.8
**Others**					
Caffeine	194	58–08–2	19.5	10.1	13.0
Phenethyl alcohol	122	60–12–8	18.3	5.6	11.2
Furfural	96	98–01–1	18.6	14.9	5.1
Methyl-2-furoate	126	611–13–2	17.4	6.9	9.7
Decanoic acid	172	334–48–5	16.2	4.2	8.3

**Table 4:** Analysis of red wine using SPE followed by GCxGC-TOF/MS.

Compound^a^	^1^t_R_^b^ (s)	^2^t_R_^b^ (s)	(%)^c^
Furan	470	1.23	0.22
Dimethylamine	480	1.41	0.20
Pentanoic acid	505	4.00	0.12
Propyl propanedioic acid	510	0.60	0.29
Monoethanolamine	560	2.33	0.32
1-Methyl-2-pyrrolidinone	590	3.61	0.48
2-Methoxyphenol	645	1.69	0.11
Phenylethyl alcohol	670	3.34	28.51
Octanoic acid	730	1.92	1.48
Ethyl hydrogen succinate	730	2.91	10.57
Butanedioic acid diethyl ester	735	2.63	10.46
Octanoic acid ethyl ester	755	1.17	0.21
2-Oxopentanedioic acid	780	2.20	0.18
4-Ethylphenyl acetate	780	3.34	11.34
2-Methoxyresorcinol	835	3.82	0.12
Hydroquinone	845	4.19	0.15
Resorcinol	855	4.08	0.13
2-Methoxy-4-vinylphenol	885	3.47	0.26
Decanoic acid	925	2.01	0.14
2,6-Dimethoxyphenol	925	4.07	2.79
4-Hydroxybenzene ethanol	995	4.56	2.59
Levoglucosan	1060	1.02	24.91
3-Hydroxy-4-methoxybenzoic acid	1120	4.50	0.27
1,3,5-Benzenetriol	1165	0.43	0.84
epi-Inositol	1235	4.66	0.14
1H-Indole-3-ethanol	1295	1.13	1.33
p-Hydroxycinnamic acid ethyl ester	1335	4.78	0.33
Unknown			1.51
**Total**			100.00

a As identified by GCxGC-TOF-MS software; names according to NIST mass spectral library.

b ^1^*t_R_* and ^2^*t_R_*, retention times in the first and second dimensions, respectively

c Percentage of each component is calculated as peak area of analyte divided by peak area of total ion chromatogram.

**Table 5:** Analysis of tap water using SPE followed by GCxGC-TOF/MS.

Compound^a^	^1^t_R_^b^ (s)	^2^t_R_^b^ (s)	(%)^c^
Butanoic acid	350	3.82	0.42
Propanal	360	4.96	0.16
Hexanal	365	3.98	2.19
2-Methyl-2-propanol	370	4.31	9.38
2-Hydroxy-3-pentanone	375	4.26	0.13
4-Hydroxy-2-pentanone	380	4.55	0.19
Furfural	390	4.97	0.61
2-Furanmethanol	405	4.73	0.22
Pentanoic acid	415	4.14	0.68
1,1-Hexylenedioxybutane	420	4.12	1.35
Dimethylamine	425	4.35	0.37
2-Heptanone	430	4.29	0.50
1,3-Dihydroxy-2-propanone	435	1.09	2.56
Heptanal	440	4.31	0.50
2(5H)-Furanone	460	1.98	0.55
2,5-Hexanedione	470	1.69	0.75
Benzaldehyde	505	1.70	3.88
Hexanoic acid	515	4.33	0.14
à-Methylstyrene	515	4.85	0.43
(E)-2-Nonene	530	1.32	0.16
1,2,3-Trimethylbenzene	530	4.69	1.42
2-Propen-1-ol	535	2.55	4.39
Octanal	535	4.53	0.32
2H-Pyran-2,6(3H)-dione	540	1.11	0.21
1-(2-Methoxypropoxy)-2-propanol	545	4.80	0.32
3-Methyl-4-Hexen-2-one	550	1.10	0.16
2-Ethyl-1-hexanol	570	4.40	1.09
1-Methyl-2-pyrrolidinone	580	2.15	17.94
Benzyl alcohol	585	1.91	1.47
Benzeneacetaldehyde	585	2.12	0.75
1-Nitrosopiperidine	590	1.27	0.77
2-Methylphenol	605	1.48	0.18
(E)-2-Octenal	605	4.79	0.34
Acetophenone	610	1.15	4.29
4-Methyl-à-oxo-benzeneacetic acid	625	1.09	0.37
2,5-Furandicarboxaldehyde	630	2.17	0.18
4-Methylbenzaldehyde	640	1.89	1.73
1-Methyl-2-piperidinone	645	1.26	0.23
1-Methyl-2,5-pyrrolidinedione	645	2.81	0.79
3-Buten-2-ol	655	2.53	0.41
Nonanal	655	4.57	0.98
Benzoic acid	720	1.72	0.43
(E)-3-Dodecene	750	3.89	1.40
Dodecane	755	3.84	0.14
Naphthalene	760	1.05	0.42
1,2-Benzenediol	760	1.14	0.18
2-Decen-1-ol	765	4.61	0.16
5-(Hydroxymethyl)-2-furancarboxaldehyde	795	2.41	3.83
3-Methyl-1,2-benzenediol	860	1.15	0.13
Tetrahydro-2H-pyran-2-methanol	865	4.39	0.18
Phenylethanolamine	880	1.81	0.28
1,2,3-Benzenetriol	950	1.84	0.20
Dodecanal	970	4.59	1.19
Diphenyl ether	980	1.22	1.37
1-Dodecanethiol	1095	4.52	0.12
Limonene	1120	1.14	0.60
1-Methylethyl ester dodecanoic acid	1165	4.35	0.40
(Z,Z)-à-Farnesene	1165	4.72	0.80
Dodecyl acrylate	1220	4.55	1.92
Hexadecane	1225	3.92	0.31
(E)-4-Dodecene	1405	2.78	0.42
Undecanoic acid	1425	4.75	0.85
2-Methylheptadecane	1525	4.02	0.69
Hexadecanoic acid butyl ester	1580	4.54	0.38
Oxalic acid allyl hexadecyl ester	1665	4.30	1.46
Heptacosane	1715	4.07	6.19
(1-Methylethyl)-cyclohexane	1770	4.46	0.31
2,2′-Methylenebis[6-(1,1-dimethylethyl)-4-ethyl-phenol	1795	1.42	2.22
Pentadecanal	1895	3.09	0.48
Unknown			9.43
**Total**			100.00

a As identified by GCxGC-TOF-MS software; names according to NIST mass spectral library.

b ^1^*t_R_* and ^2^*t_R_*, retention times in the first and second dimensions respectively

c Percentage of each component is calculated as peak area of analyte divided by peak area of total ion chromatogram.
