# Supplementary figures and images for: Sustainable bio-based solid phase extraction adsorbent for the determination of various types of organic compounds
Source: Turk J Chem. 2023 Nov 27;48(1):36–49. doi: 10.55730/1300-0527.3637 (PMC10965176; doi:10.55730/1300-0527.3637)

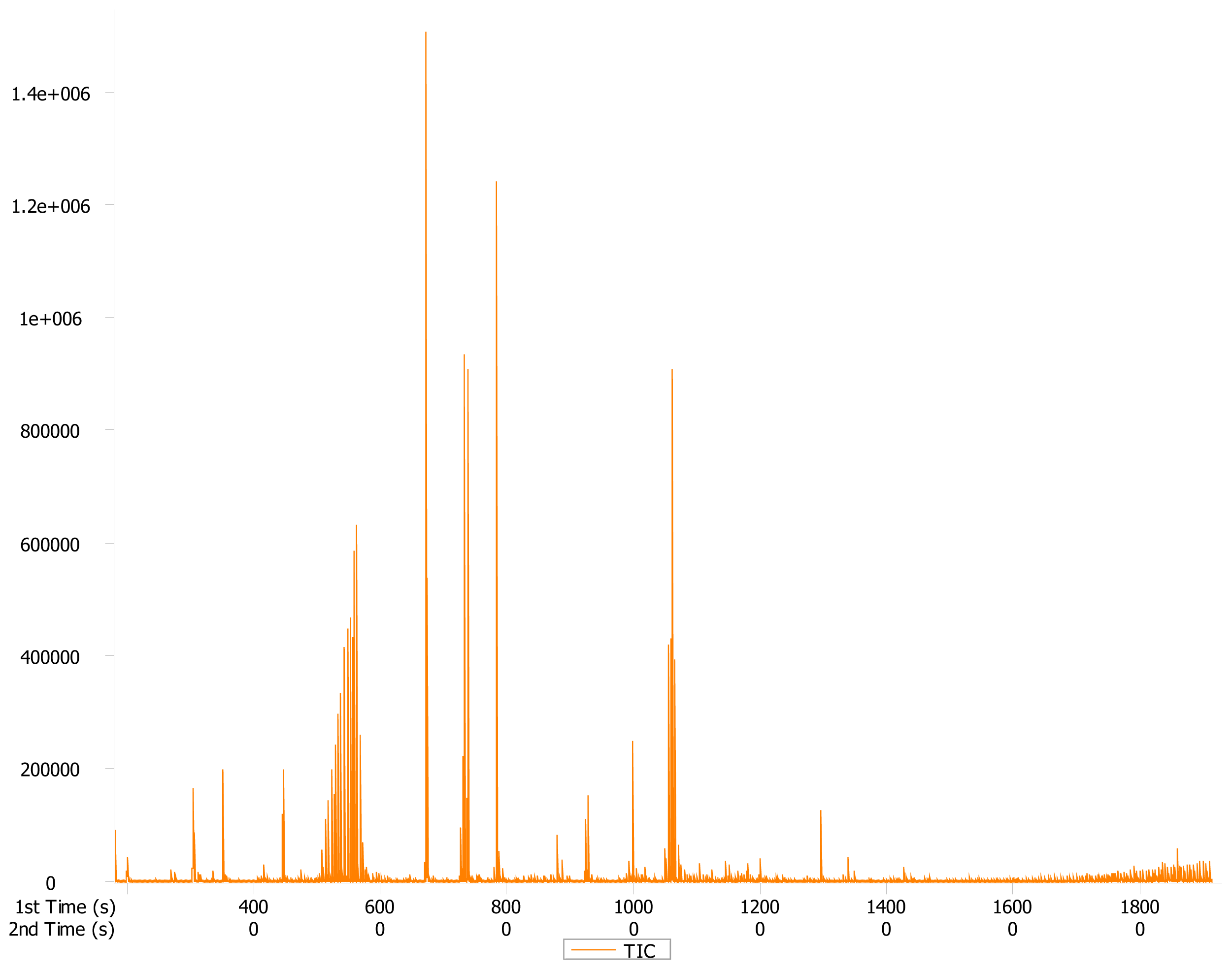

Supplement: Figure S1 — GCxGC-TOF/MS chromatogram of red wine after Starbon A800 extraction. [file tjc-48-01-0036s1.tif]

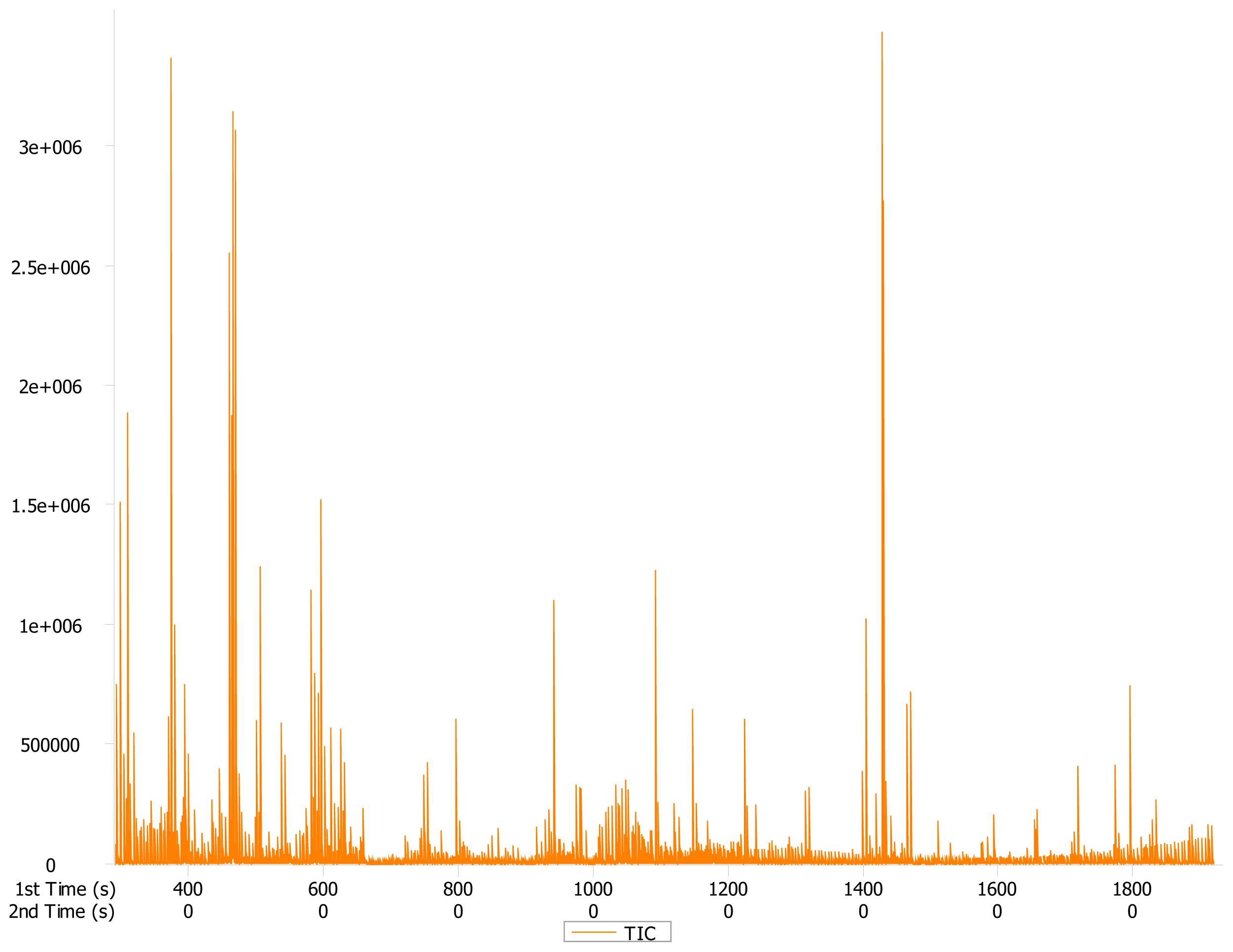

Supplement: Figure S2 — GCxGC-TOF/MS chromatogram of tap water after Starbon A800 extraction. [file tjc-48-01-0036s2.tif]
